# The Use of Ketamine and Dexmedetomidine in Cesarean Section: A Narrative Review of Clinical Applications and Safety Considerations

**DOI:** 10.5812/aapm-163063

**Published:** 2025-08-25

**Authors:** Thaer Kareem Oleiwi Atabi, Ali Jabbari, Somayeh Ghorbani Gholiabad, Hamzeh Bader Gazal, Ali Movafegh

**Affiliations:** 1 Department of Anesthesiology and Critical Care, Faculty of International, Golestan University of Medical Sciences, Gorgan, Iran; 2 Department of Anesthesiology and Critical Care, Ischemic Disorders Research Center, Faculty of Medicine, Golestan University of Medical Sciences, Gorgan, Iran; 3 Cancer Research Center, Golestan University of Medical Sciences, Gorgan, Iran; 4 Department of Anesthesia and Intensive Care, Al Zahra'a Teaching Hospital, Kut, Iraq; 5 Department of Anesthesiology, School of Medicine, Tehran University of Medical Sciences, Tehran, Iran

**Keywords:** Cesarean Section, Anesthesia, Ketamine, Dexmedetomidine, Pharmacogenetics

## Abstract

**Context:**

Cesarean section (CS) represents a prevalent surgical intervention necessitating meticulous and efficacious anesthetic administration to optimize maternal and neonatal well-being. This narrative review aims to comprehensively examine the pharmacological properties, clinical applications, and safety considerations associated with the utilization of ketamine and dexmedetomidine as anesthetic agents within the context of CS.

**Evidence Acquisition:**

A structured literature search was performed across PubMed, Scopus, and Web of Science databases using key terms including 'Ketamine', 'dexmedetomidine', 'cesarean section', 'anesthesia', and 'pharmacogenetics'. Inclusion criteria were applied to guide the selection of relevant studies for a narrative synthesis. Data collection involved identifying information pertinent to anesthesia types (neuraxial/general), administration routes [intravenous (IV)/intrathecal], dosages, and maternal and neonatal outcomes for a qualitative summary. This narrative review did not include a formal risk of bias assessment or quantitative meta-analysis.

**Results:**

A narrative synthesis of the identified literature indicates that ketamine, recognized for its dissociative anesthetic characteristics and significant analgesic potency, contrasts with dexmedetomidine, which offers distinct sedative and analgesic actions while exhibiting limited respiratory depressant effects. The concurrent administration of these two pharmacological agents holds the potential for synergistic interactions, potentially leading to improved patient outcomes through mechanisms such as reduced opioid requirements, enhanced hemodynamic stability, and minimized postoperative adverse events.

**Conclusions:**

This review underscores the potential for synergistic effects between ketamine and dexmedetomidine in enhancing both analgesic efficacy and hemodynamic stability during CS. Furthermore, it examines the safety profiles associated with this combination and considers relevant pharmacogenetic factors.

## 1. Introduction

A cesarean section (CS) is a common surgical intervention in obstetrics, frequently indicated by various maternal and fetal conditions such as fetal distress, cephalopelvic disproportion, placenta previa, and specific maternal medical issues (1). This procedure poses distinct challenges for anesthesia providers, necessitating a meticulous balance between ensuring maternal well-being and safeguarding fetal health. The fundamental objectives of anesthesia during CS are to achieve adequate surgical anesthesia, ensure maternal comfort, and minimize fetal exposure to anesthetic agents, all while concurrently managing the substantial physiological adaptations inherent to pregnancy (2). The profound physiological alterations induced by pregnancy significantly influence anesthetic management. The physiological adaptations during pregnancy encompass an augmentation in blood volume, cardiac output, and heart rate. Furthermore, the gravid uterus can exert aortocaval compression, potentially resulting in supine hypotension syndrome (3). Consequently, meticulous patient positioning and vigilant hemodynamic surveillance are essential. The cephalad displacement of the diaphragm, coupled with heightened oxygen consumption and a reduction in functional residual capacity, renders pregnant individuals susceptible to rapid oxygen desaturation during airway management (4). Delayed gastric emptying and reduced lower esophageal sphincter tone elevate the susceptibility to aspiration pneumonitis (5). Furthermore, increased progesterone and estrogen levels significantly impact the pharmacokinetic and pharmacodynamic profiles of anesthetic agents (6). The physiological state of pregnancy also induces a hypercoagulable condition, thereby augmenting the potential for thromboembolic events (7). Anesthesia methods for CS need to consider these hemodynamic and respiratory alterations, reduce pain after surgery, and prevent negative complications like hypotension, respiratory depression, and maternal anxiety, all ensuring the best possible oxygenation and health for the fetus. Regional anesthetic techniques, encompassing spinal and epidural modalities, represent the favored approach for planned CSs owing to their established safety profile. This preference stems from their capacity to maintain maternal consciousness and limit fetal exposure to pharmacological agents (8). While spinal anesthesia is characterized by its swift onset and profound sensory and motor blockade, epidural anesthesia affords greater adaptability for both intraoperative management and subsequent postoperative pain relief (9). Combined spinal-epidural anesthetic techniques provide the advantages inherent to both modalities. General anesthesia is limited to cases of emergent CS or when regional anesthesia is deemed unsuitable. The administration of general anesthesia necessitates rapid sequence induction and endotracheal intubation to minimize the potential for pulmonary aspiration. In comparison to regional anesthesia, general anesthesia is associated with an elevated risk of maternal complications and fetal depression (10).

Although regional anesthesia is the established standard for CS, ongoing limitations include opioid-induced respiratory depression, hypotension, and the inherent variability in individual responses to anesthetic agents (11). Ketamine and dexmedetomidine present distinct benefits, such as the hemodynamic support and opioid-sparing characteristics of ketamine, and the sedative effects of dexmedetomidine without causing respiratory depression (12, 13). Nevertheless, there are existing gaps in the evidence concerning their ideal application, dosage regimens, and the impact of pharmacogenetic factors. This review aims to synthesize the current body of evidence to inform clinical decision-making in this context.

Ketamine, functioning as an N-methyl-D-aspartate (NMDA) receptor antagonist, induces rapid analgesia, amnesia, and dissociative anesthesia. Its capacity to elevate blood pressure renders it particularly advantageous in the management of hemodynamically unstable patients (14). However, despite its efficacy, ketamine administration carries a risk of psychomimetic adverse effects, which can be mitigated through the use of low dosages (15). Dexmedetomidine, an alpha-2 (α2)-adrenergic receptor agonist, provides both sedation and analgesia with a notable absence of significant respiratory depression, which can be advantageous for maintaining hemodynamic stability during procedures such as endotracheal intubation. However, its administration may be associated with the development of bradycardia and hypotension, particularly in patients experiencing hypovolemia (16). Combining these agents can yield synergistic effects, thereby enable the administration of lower dosages and consequently minimize individual adverse effects. Specifically, dexmedetomidine can attenuate the psychomimetic effects associated with ketamine, while ketamine enhances the analgesic properties of dexmedetomidine (17). This synergistic approach enhances patient well-being, diminishes the requirement for opioid analgesics, and promotes hemodynamic stability, which is of critical importance in the context of CSs. Ketamine and dexmedetomidine are critical agents in enhancing postoperative analgesia and mitigating side effects associated with opioid administration. This review investigates the utility of ketamine and dexmedetomidine as adjunctive therapies in the context of CS anesthesia. The scope of this analysis encompasses their respective mechanisms of action, pharmacokinetic profiles, clinical applications, safety considerations, and the potential for synergistic interactions when co-administered. 

## 2. Literature Search Strategy

A structured and comprehensive literature search was performed across PubMed, Scopus, and Web of Science databases employing the following keywords: 'Ketamine', 'dexmedetomidine', 'cesarean section', 'anesthesia', and 'pharmacogenetics'. This search aimed to provide a broad overview for a narrative synthesis, rather than a systematic review or meta-analysis. The search was conducted from the inception of each database up to March 2025. Only articles published in English were considered for inclusion. The inclusion criteria for studies encompassed randomized controlled trials (RCTs), non-randomized controlled clinical trials, cohort studies, case-control studies, meta-analyses, systematic reviews, case reports, and pharmacogenetic association studies that assessed the utilization of ketamine and/or dexmedetomidine in anesthesia for CS. Studies that were not relevant to human CS or the specified anesthetic agents (ketamine and dexmedetomidine), or those focusing purely on basic science research without direct clinical implications in CS, were excluded from this narrative review.

Data extraction procedures prioritized the collection of information pertaining to the anesthesia modality (neuraxial/general), administration routes [intravenous (IV)/intrathecal], administered dosages, and both maternal and neonatal outcomes. As this is a narrative review, a formal preferred reporting items for systematic reviews and meta-analyses (PRISMA) flow diagram was not generated, nor was the study prospectively registered. Moreover, given the nature of this review as a narrative synthesis, a formal assessment of the risk of bias for individual studies was not conducted.

## 3. Pharmacology of Ketamine and Dexmedetomidine; Mechanism of Action, Pharmacokinetic Profile, and Pharmacodynamic Effects

Ketamine, a distinct dissociative anesthetic agent, primarily exerts its pharmacological action through non-competitive antagonism of the NMDA receptor (18). The NMDA receptor, a subtype of glutamate receptors, is critically involved in synaptic plasticity, nociception, and the development of central sensitization, a key mechanism underlying chronic pain conditions (19). By impeding the function of the NMDA receptor, ketamine disrupts the transmission of pain signals within the central nervous system, thereby effectively diminishing pain perception (20). However, the pharmacological influence of ketamine extends beyond its role as an NMDA receptor antagonist. It interacts with a diverse array of other receptor systems, playing a role in its intricate and multifaceted clinical effects. This substance exerts its effects through interactions with several receptor types. Notably, it engages α-amino-3-hydroxy-5-methyl-4-isoxazolepropionic acid (AMPA) receptors, which may underlie its rapid antidepressant properties. It also binds to opioid receptors, particularly the mu (μ) subtype, contributing to its analgesic effects. Furthermore, it interacts with muscarinic receptors, leading to anticholinergic effects, such as elevated heart rate and bronchodilation; it finally modulates monoaminergic systems, encompassing dopamine, norepinephrine, and serotonin, which are implicated in its psychotropic actions (21-23). Ketamine exhibits a pharmacokinetic profile characterized by its rapid absorption via multiple routes of administration, including IV, intramuscular (IM), and intranasal (IN) (24). While IV administration facilitates the most immediate onset of action, IM and IN routes present viable alternatives for administration when IV access is not feasible. Following its systemic uptake, ketamine undergoes significant hepatic biotransformation, primarily mediated by the cytochrome P450 (CYP) enzymatic system, with the CYP3A4 isoenzyme identified as the principal catalyst (25). This metabolic process yields several compounds, including norketamine, which also exhibits a degree of pharmacological activity (26). Ketamine is characterized by a relatively brief elimination half-life, typically ranging from 2 to 3 hours. This pharmacokinetic property results in a rapid onset and offset of its pharmacological action. Such a profile is particularly advantageous in medical procedures of short duration, including CS, where the precise and timely management of anesthetic depth is of paramount importance (27). The metabolic profile of ketamine can be influenced by several factors, including genetic polymorphisms affecting CYP enzymes, hepatic function, and concurrent administration of other medications (28). Ketamine is well-established for its significant analgesic properties, which are evident even when administered at dosages below those required for general anesthesia. It elicits a dissociative state in patients, a condition marked by profound analgesia, amnesia, and a feeling of being disconnected from their surroundings (24). When administered at low dosages during CS, ketamine can contribute to hemodynamic stability through the augmentation of sympathetic tone. This effect may be particularly advantageous in patients presenting with pre-eclampsia or those susceptible to hypotension induced by other anesthetic agents (29). Conversely, the administration of ketamine at higher doses can precipitate excessive sympathetic nervous system stimulation, potentially leading to adverse cardiovascular events such as hypertension, tachycardia, and an elevation in myocardial oxygen demand (30). 

Dexmedetomidine, a highly selective agonist of the α2-adrenergic receptor, exerts its pharmacodynamic actions through the activation of these receptors. These receptors are predominantly located within the locus coeruleus, a nucleus in the brainstem critically involved in the modulation of arousal states and the activity of the sympathetic nervous system (31). Activation of α2-adrenergic receptors within the locus coeruleus leads to a reduction in norepinephrine release. This consequently diminishes sympathetic outflow, resulting in pharmacological effects such as sedation, anxiolysis, and analgesia (32). Furthermore, dexmedetomidine exerts its analgesic action at the spinal level by activating α2-receptors in the dorsal horn. This activation inhibits the transmission of nociceptive signals, thereby augmenting its analgesic properties (33). Unlike non-selective adrenergic receptor agonists, which can activate both α1 and α2 subtypes, dexmedetomidine exhibits high selectivity for α2-receptors. This pronounced selectivity mitigates adverse effects such as vasoconstriction and tachycardia, rendering it a preferred agent in specific clinical scenarios (34). Typically administered via the IV route, dexmedetomidine allows for fine-tuned regulation of its plasma concentration. The compound undergoes hepatic biotransformation via glucuronidation and CYP enzymes (35). Its pharmacokinetic profile is characterized by a rapid distribution phase followed by a more protracted elimination phase. The elimination half-life of the drug is within the range of 2 to 3 hours, and its clearance rate is susceptible to alterations in the presence of hepatic or renal dysfunction, thereby necessitating meticulous dosage adjustments in affected patient populations. Dexmedetomidine elicits a distinctive sedative state characterized as "awake sedation", wherein patients exhibit a calm and cooperative demeanor while retaining the capacity for facile arousal (36). This particular attribute renders it especially advantageous in medical interventions necessitating patient collaboration, such as the administration of regional anesthesia. In contrast to other sedative agents, such as benzodiazepines and opioids, dexmedetomidine exhibits a profile of minimal respiratory depression, rendering it a potentially safer option for patients with heightened susceptibility to respiratory compromise (37). Furthermore, dexmedetomidine offers hemodynamic advantages by attenuating sympathetic nervous system activity and promoting cardiovascular stability during surgical procedures. The capacity of this agent to decrease heart rate and blood pressure may offer specific benefits for patients diagnosed with hypertension or tachycardia (38) (Table 1). In the context of CS anesthesia, ketamine is typically administered via the IV (0.25 - 0.5 mg/kg for analgesia; 1 - 2 mg/kg for induction). It is also used off-label as an adjunct via the epidural route. Dexmedetomidine is administered via the IV as a bolus (0.5 - 1 μg/kg; 0.2 - 0.7 μg/kg/h infusion) or intrathecally (5 - 10 μg) (39, 40).

**Table 1.** Pharmacology of Ketamine and Dexmedetomidine

Feature	Ketamine	Dexmedetomidine	Clinical Implication
**Mechanism of action**	Non-competitive NMDA receptor antagonism; interacts with opioid, AMPA, and muscarinic receptors.	Selective α2-adrenergic receptor agonist; reduces norepinephrine release in the locus coeruleus.	Ketamine reduces central sensitization and opioid needs; dexmedetomidine stabilizes hemodynamics via sympatholysis.
**Routes of administration**	IV (0.25 - 0.5 mg/kg analgesia; 1 - 2 mg/kg induction), epidural (off-label)	IV (0.5 - 1 μg/kg bolus; 0.2 - 0.7 μg/kg/h infusion); intrathecal (5 - 10 μg, limited evidence)	Ketamine IV is preferred for rapid onset; dexmedetomidine IV/intrathecal balances sedation and analgesia.
**Pharmacokinetics**	Rapid absorption; hepatic metabolism (CYP3A4); half-life: 2 - 3 hours	Hepatic glucuronidation/CYP450; half-life: 2 - 3 hours; caution in hepatic/renal impairment.	Dose adjustments are needed in hepatic dysfunction for both drugs.
**Pharmacodynamic effects**	Dissociative anesthesia, analgesia, and sympathetic stimulation (↑ BP/HR at high doses)	Sedation, anxiolysis, and minimal respiratory depression; ↓ HR/BP	Ketamine benefits hypotension; dexmedetomidine risks bradycardia.
**Advantages in CS**	Opioid-sparing, hemodynamic support in pre-eclampsia/obesity	Sedation without respiratory depression reduces opioid needs.	Synergistic when combined: Ketamine offsets dexmedetomidine-induced bradycardia.
**Disadvantages/side effects**	Psychomimetic effects (20%), nausea/vomiting (15 - 30%), and hypertension (high doses)	Bradycardia (10 - 15%), hypotension (5 - 10%), and dry mouth	Low-dose ketamine (0.1 - 0.3 mg/kg) minimizes side effects.

Abbreviations: NMDA, N-methyl-D-aspartate; AMPA, α-amino-3-hydroxy-5-methyl-4-isoxazolepropionic acid; α2, alpha-2; IV, intravenous; CYP, cytochrome P450; CS, cesarean section.

## 4. Ketamine and Dexmedetomidine in Cesarean Section Anesthesia

As an adjunct to general anesthesia during CS, ketamine presents a multimodal strategy for enhancing patient outcomes (41). Its primary pharmacological benefit is the reduction of opioid requirements, which is clinically significant due to the potential for opioid-induced respiratory depression in both the mother and the adverse neonatal effects. Ketamine, as a non-competitive antagonist at the NMDA receptor, disrupts the process of central sensitization. This action leads to a reduction in the perception of acute surgical pain and mitigates the development of chronic postoperative pain (40, 42). Consequently, the significant analgesic efficacy of ketamine allows for a considerable decrease in the required dosages of both intraoperative and postoperative opioids, thereby minimizing their associated adverse effects. Beyond its established mechanism of action as an NMDA receptor antagonist, ketamine also exhibits interactions with opioid receptors, thereby potentiating analgesic outcomes when administered concurrently with opioid medications (43). Clinically, ketamine finds its primary application as an adjunctive agent in the context of general anesthesia (IV) or epidural analgesia. In contrast, dexmedetomidine is preferentially employed for sedation during neuraxial anesthesia (IV) or as a component of balanced general anesthesia regimens. Notably, RCTs have demonstrated that the administration of low-dose ketamine (0.1 to 0.3 mg/kg IV) can reduce postoperative pain scores by 30 - 50% (44). Similarly, studies, including RCTs, have shown that dexmedetomidine (0.5 μg/kg IV, prior to surgical incision) can decrease intraoperative opioid requirements by approximately 40% (39).

Maintaining hemodynamic stability is of critical importance during CS, especially in individuals with pre-eclampsia, obesity, or other coexisting medical conditions that elevate their susceptibility to hypotension (45). Low-dose ketamine exerts a stimulatory effect on the sympathetic nervous system, consequently leading to elevations in heart rate, blood pressure, and cardiac output (46). The aforementioned effect demonstrates particular utility in individuals with pre-eclampsia, a condition frequently characterized by reduced intravascular volume and compromised vasomotor tone. Furthermore, in obese patients, who may exhibit altered pharmacokinetic and pharmacodynamic profiles with respect to anesthetic agents, the hemodynamic properties of ketamine can contribute to the maintenance of blood pressure during both the induction of anesthesia and subsequent surgical stimulation (47). Low-dose ketamine has demonstrated efficacy in diminishing the requirement for supplementary analgesic medications during and after surgical procedures, consequently leading to a more stable and effective management of pain. The underlying mechanisms responsible for this reduction in rescue analgesic use are likely complex and involve several pharmacological actions of ketamine, including its antagonism of NMDA antagonism, interactions with opioid receptors, and potential anti-inflammatory properties (48).

The primary application of dexmedetomidine in the context of CS anesthesia is to induce procedural sedation, particularly when administered in conjunction with regional anesthetic techniques, such as spinal or epidural anesthesia (49). Dexmedetomidine, through the activation of α2-adrenergic receptors within the locus coeruleus, diminishes the release of norepinephrine. This pharmacological action results in a state of tranquil sedation that is not typically associated with clinically significant respiratory depression. This characteristic is of critical importance during CS procedures, as maternal respiratory compromise can negatively impact fetal oxygenation (50). Clinical experience and observational studies suggest that dexmedetomidine is efficacious in diminishing anxiety, a prevalent condition among women undergoing CS, and in enhancing patient compliance during the procedure, thereby facilitating a more seamless surgical course. Its distinctive property of inducing "awake sedation" enables patients to maintain responsiveness to verbal commands while experiencing a reduction in anxiety and discomfort (51).

Academically, dexmedetomidine exhibits noteworthy cardiovascular stabilizing properties, particularly advantageous for patients with pre-existing cardiac conditions. Its mechanism of action, involving the reduction of sympathetic nervous system outflow, effectively mitigates the potential for intraoperative hypotension and bradycardia. These hypotensive and bradycardic events can be precipitated by surgical stimuli or the administration of anesthetic agents (52). This attenuation of hemodynamic instability is especially critical in individuals with compromised cardiac function, who demonstrate heightened susceptibility to such fluctuations. The resultant decrease in heart rate and blood pressure typically manifests gradually and is generally well-tolerated, contingent upon the maintenance of adequate patient hydration. Furthermore, dexmedetomidine's capacity to preserve hemodynamic stability concurrently with minimal respiratory depression renders it a clinically significant adjunct in CS anesthesia (53).

The combined application of ketamine and dexmedetomidine presents a potentially advantageous strategy for optimizing both analgesia and sedation in the context of CS (44). The pronounced analgesic effects of ketamine, when integrated with the sedative and hemodynamically stabilizing properties of dexmedetomidine, culminate in a well-rounded anesthetic profile. This synergistic combination may facilitate the administration of lower individual dosages, thereby mitigating the potential for agent-specific adverse effects. Dexmedetomidine has the capacity to mitigate the psychomimetic sequelae associated with ketamine administration, including phenomena such as hallucinations and nightmares, which can emerge as a significant clinical consideration at elevated dosages. Conversely, ketamine may potentiate the analgesic properties of dexmedetomidine, thereby contributing to more efficacious pain management (39). To mitigate potential adverse effects while optimizing therapeutic outcomes, meticulous dose titration of these agents is paramount. Notably, both ketamine and dexmedetomidine are associated with the risk of inducing hypotension, a concern that is particularly pronounced in patients with hypovolemia. The concurrent administration of these two medications may also synergistically augment this hypotensive effect (54). Consequently, rigorous surveillance of hemodynamic variables, encompassing blood pressure, heart rate, and oxygen saturation, is of paramount importance. While respiratory depression is an infrequent occurrence with dexmedetomidine monotherapy, its potential arises with elevated dosages of ketamine or its concurrent administration with other respiratory depressant agents. Emergence agitation, a recognized adverse effect of ketamine, can be mitigated through the application of reduced dosages and the concomitant use of dexmedetomidine (55). The determination of the optimal dosing strategy for ketamine and dexmedetomidine in the context of CS anesthesia remains an area requiring further scholarly inquiry to develop evidence-based clinical recommendations. In determining the suitable dosage and synergistic application of these pharmacological agents, a thorough evaluation of specific individual patient characteristics is essential. These factors encompass age, weight, the presence of comorbidities, and the specific anesthetic technique employed (Table 2). 

**Table 2.** Ketamine and Dexmedetomidine in Cesarean Section Anesthesia

Feature	Ketamine	Dexmedetomidine	Combination (Ketamine + Dexmedetomidine)
**Primary role in CS**	Adjunct to general anesthesia; opioid-sparing analgesia; hemodynamic support	Procedural sedation (regional anesthesia); anxiolysis; cardiovascular stability	Synergistic analgesia and sedation; combined benefits of hemodynamic stability and opioid reduction; minimization of individual side effects
**Mechanism of action benefits**	NMDA receptor antagonism (analgesia, prevention of central sensitization); sympathetic stimulation (hemodynamic support); opioid receptor interaction (enhanced analgesia)	α2-adrenergic receptor agonism (reduced norepinephrine release, spinal analgesia); minimizes sympathetic outflow (hemodynamic stability, anxiolysis)	Combined NMDA antagonism and α2 agonism; attenuation of individual side effects (dexmedetomidine minimizes ketamine's psychomimetic effects); enhanced analgesic and sedative profile
**Hemodynamic effects**	Increased heart rate, blood pressure, and cardiac output (low doses); beneficial in pre-eclampsia, obesity, and hypotension.	Reduced sympathetic tone; minimized risk of hypotension and bradycardia; gradual reduction in heart rate and blood pressure; stable cardiovascular parameters	Potentially additive hypotensive effects; requires careful hemodynamic monitoring; combined effect on cardiovascular stability needs careful titration.
**Analgesic effects**	Potent analgesic; reduces intraoperative and postoperative opioid requirements; effective pain control in pre-eclampsia and obese patients	Sedation and analgesia without significant respiratory depression; reduces opioid needs	Enhanced analgesia allows for lower doses of each drug and optimized pain control.
**Sedative effects**	Dissociative anesthesia; amnesia	"Awake sedation"; anxiolysis; improved patient cooperation	Balanced sedation; attenuation of ketamine's emergence agitation.
**Respiratory effects**	May cause respiratory depression, particularly at higher doses; risk of laryngospasm.	Minimal respiratory depression; safe in patients with compromised respiratory function	Careful monitoring of respiratory function is crucial; the combination can potentiate respiratory depression.
**Adverse effects considerations**	Emergence phenomena, hypertension/tachycardia, nausea/vomiting	Hypotension, bradycardia, dry mouth, and nausea	Potentially increased risk of hypotension, bradycardia, and respiratory depression; requires careful titration and monitoring.
**Patient population benefits**	Beneficial in pre-eclampsia, obese patients, and those at risk of hypotension.	Valuable in patients with underlying cardiovascular conditions and anxiety.	Optimized outcomes for patients with comorbidities; reduced overall side effects
**Dosing considerations**	Low-dose ketamine preferred; careful titration based on patient factors	Careful dosing based on patient factors, especially in hepatic or renal impairment	Careful titration is crucial; individual patient factors must be considered; further research is needed to establish optimal dosing guidelines.
**Clinical use**	Often used in emergency cases where hemodynamic support is needed or rapid sequence inductions are required.	Used most often as an adjunct to regional anesthesia, or as a component of TIVA (total IV anesthesia).	Used when synergistic effects are desired to achieve analgesia, sedation, and hemodynamic control with reduced side effects.

Abbreviations: CS, cesarean section; NMDA, N-methyl-D-aspartate; α2, alpha-2; IV, intravenous.

## 5. Pharmacogenetics and Personalized Medicine

The nascent field of pharmacogenetics presents a substantial opportunity to transform anesthetic practices, particularly concerning the application of ketamine and dexmedetomidine in the context of CS. Genetic polymorphisms, which represent variations in DNA sequences, can significantly modulate the way individuals metabolize and respond to these anesthetic drugs. This genetic variability ultimately contributes to inter-patient differences in both the effectiveness and potential toxicity of these agents (56). Understanding these individual genetic differences can lead to more personalized anesthetic approaches, optimizing outcomes and minimizing adverse effects.

Ketamine undergoes significant hepatic metabolism, primarily facilitated by the CYP enzyme system, with CYP3A4 being a key enzyme involved. Genetic polymorphisms in CYP3A4, as well as in other CYP enzymes that metabolize ketamine (such as CYP2B6 and CYP2C9), can lead to variations in enzyme activity (25, 57). Individuals possessing genetic variants that result in increased enzyme activity may exhibit accelerated ketamine metabolism. This rapid metabolism can culminate in lower circulating plasma concentrations of the drug, potentially resulting in subtherapeutic effects. Conversely, individuals exhibiting diminished enzymatic activity may experience a decelerated metabolic rate, consequently leading to elevated plasma drug concentrations and a heightened susceptibility to adverse effects, such as protracted psychomimetic effects or cardiovascular instability (58). For instance, single-nucleotide polymorphisms (SNPs) within the CYP3A4 gene can modulate its expression and catalytic efficiency, thereby influencing the clearance of ketamine (25). 

Similarly, dexmedetomidine, an α2-adrenergic receptor agonist, exerts its sedative, analgesic, and hemodynamic effects through interactions with adrenergic receptors, particularly the α2A-adrenergic subtype. Polymorphisms within the genes encoding these receptors, such as variations in the alpha-2A adrenergic receptor (ADRA2A) gene, can modulate receptor sensitivity and downstream signaling cascades, thereby influencing the pharmacological response to dexmedetomidine. Individuals exhibiting specific ADRA2A genotypes may demonstrate variable responses to dexmedetomidine, necessitating tailored dosage adjustments to achieve the desired clinical outcomes (59). Notably, particular polymorphisms within the ADRA2A gene have been correlated with a spectrum of sedation levels and hemodynamic changes following dexmedetomidine administration (60). Genetic variations influencing responsiveness to both ketamine and dexmedetomidine are compiled in Table 3. 

**Table 3.** Genetic Polymorphisms Affecting Ketamine and Dexmedetomidine

Drug; Gene	Polymorphism/Variant	Potential Impact	Clinical Implications
**Ketamine**			
CYP3A4	SNPs (CYP3A4*1B, CYP3A4*22)	Variable enzyme activity (increased or decreased); altered ketamine metabolism	Dose adjustments are needed to achieve therapeutic levels; risk of subtherapeutic effects or toxicity
CYP2B6	SNPs (CYP2B6*6, CYP2B6*9)	Variable enzyme activity; altered ketamine metabolism	Similar to CYP3A4, potential for altered ketamine clearance
CYP2C9	SNPs (CYP2C9*2, CYP2C9*3)	Variable enzyme activity; altered ketamine metabolism	Similar to CYP3A4, potential for altered ketamine clearance
OPRM1 (μ-opioid receptor)	SNPs (A118G)	Altered opioid receptor sensitivity; variable analgesic response to ketamine	Variability in ketamine's analgesic efficacy; potential need for alternative analgesics
CHRM2 (muscarinic receptor)	-	Altered muscarinic receptor sensitivity	Variability in anticholinergic side effects (e.g., tachycardia, dry mouth)
**Dexmedetomidine**			
ADRA2A	SNPs (-1291 C>G, -1296 C>T, RS553668)	Altered α2A-adrenergic receptor sensitivity; variable sedative and hemodynamic response	Dose adjustments needed to achieve desired sedation and hemodynamic stability; risk of bradycardia or hypotension
ADRA2C	-	Altered α2C-adrenergic receptor sensitivity	Potential variations in sedative and hemodynamic effects
CYP2A6	-	Variation in the enzymes that metabolize dexmedetomidine.	Altered dexmedetomidine clearance and potential for toxicity
UGT1A4	-	Variation in the enzymes that metabolize dexmedetomidine.	Altered dexmedetomidine clearance and potential for toxicity

Abbreviations: SNP, single-nucleotide polymorphism; CYP, cytochrome P450; α2, alpha-2; μ, mu; ADRA2A, alpha-2A adrenergic receptor.

Understanding the inherent genetic diversity among individuals holds the potential to revolutionize anesthetic practices by enabling clinicians to personalize drug administration. This tailored approach aims to optimize therapeutic outcomes while mitigating the incidence of adverse effects. The application of pharmacogenetic analysis, which involves the examination of an individual's genetic makeup to identify pertinent polymorphisms, could facilitate more precise dose adjustments for agents such as ketamine and dexmedetomidine. By identifying patients possessing genetic variations that predispose them to altered drug metabolism or receptor sensitivity, clinicians can individualize pharmacological interventions by adjusting the dosage and route of administration of these agents to optimize the therapeutic window (61). In the context of ketamine, this could involve tailoring the dose based on an individual's CYP enzyme activity. This personalized approach aims to ensure the patient receives a sufficient drug concentration to achieve the desired analgesic and amnestic effects while concurrently minimizing the occurrence of excessive adverse effects. Personalized dexmedetomidine administration could involve tailoring the dosage based on a patient's α2-adrenergic receptor genotype. This approach aims to ensure the delivery of an appropriate drug quantity to achieve the desired sedation level and hemodynamic stability while mitigating the risks of bradycardia or hypotension (62). Such individualized strategies hold promises for enhancing patient outcomes by decreasing the occurrence of adverse effects and improving the efficacy of anesthesia. The integration of pharmacogenetics into clinical anesthesiology remains nascent, necessitating additional investigation to substantiate current methodologies. Robust, large-scale clinical investigations are imperative to pinpoint the most pertinent genetic indicators influencing patient response to ketamine and dexmedetomidine. Furthermore, these studies are crucial for the formulation of evidence-based protocols that incorporate pharmacogenetic data to guide dosage administration. While routine application of pharmacogenetic testing is not yet established, genetic variations in CYP3A4 or ADRA2A may offer potential for refining dosage adjustments. For instance, individuals identified as poor metabolizers of CYP3A4 (comprising 5 - 10% of the Caucasian population) might necessitate a 20–30% reduction in ketamine dosage to mitigate the risk of prolonged sedative effects (25).

## 6. Adverse Effects and Safety Considerations

Ketamine, while recognized for its potent analgesic and anesthetic properties, is also associated with a spectrum of potential adverse effects. Notably, emergence phenomena, encompassing hallucinations, vivid dreams, and agitation, represent a common sequelae. These psychomimetic effects can induce significant distress in patients, frequently necessitating clinical intervention. The likelihood and intensity of emergence phenomena exhibit a dose-dependent relationship, with escalating dosages correlating with an increased risk profile. Strategies aimed at mitigating these undesirable effects include the administration of lower ketamine doses, the concurrent use of benzodiazepines or alternative sedative agents, and the provision of a tranquil and minimally stimulating environment during the emergence phase (63). Ketamine's impact on the cardiovascular system represents another critical consideration. Administration of the drug at elevated dosages can induce sympathetic nervous system activation, subsequently resulting in hypertension, tachycardia, and myocardial oxygen demand. These physiological alterations pose a specific risk for individuals with pre-existing cardiovascular conditions (64). In academic terms, ketamine administration is associated with an elevation in cerebral blood flow and intracranial pressure (ICP), thereby establishing it as a contraindication in patient populations presenting with head trauma or pre-existing elevated ICP. Conversely, the cardiovascular effects observed at sub-anesthetic dosages are typically minimal and may even confer therapeutic benefits in individuals experiencing hypotension (65). Furthermore, ketamine has the potential to induce adverse effects, such as nausea and vomiting, a phenomenon attributed to its interaction with the chemoreceptor trigger zone (66). Laryngospasm, a rare yet potentially critical complication, may also arise, particularly in individuals exhibiting airway hyperreactivity. Consequently, meticulous surveillance of airway patency and respiratory function is paramount (67). To mitigate these risks, precise dose titration based on the patient's clinical condition and existing comorbidities is essential.

While generally exhibiting a favorable safety profile, dexmedetomidine administration is associated with specific adverse effects stemming from its agonistic activity at α2-adrenergic receptors. The most frequently observed of these are hypotension and bradycardia. The former arises due to a reduction in sympathetic nervous system outflow, whereas the latter is mediated through both central and peripheral mechanisms. The aforementioned hemodynamic effects typically exhibit a dose-dependent relationship and may be more pronounced in patients with hypovolemia or pre-existing cardiovascular pathologies. Consequently, meticulous monitoring of blood pressure and heart rate is essential, and therapeutic interventions such as dose adjustments or the administration of fluids or vasopressors might be indicated (68). Regarding respiratory function, dexmedetomidine generally elicits minimal depression, particularly when administered within the recommended dosage range. Notwithstanding its relative safety profile, the potential for respiratory depression exists, particularly with elevated dosages or in individuals exhibiting compromised respiratory physiology, including conditions such as obstructive sleep apnea or chronic obstructive pulmonary disease. Consequently, continuous vigilance of respiratory rate and arterial oxygen saturation is paramount. Minor adverse effects associated with dexmedetomidine administration may include dry mouth, nausea, and headache. The occurrence of dry mouth is mechanistically linked to its anticholinergic properties, whereas nausea and headache are reported with lower frequency (69).

The concurrent administration of ketamine and dexmedetomidine may elicit additive or synergistic pharmacological interactions, potentially escalating the likelihood of specific adverse events. For instance, the combined use of these agents could potentiate the occurrence of hypotension and bradycardia. Consequently, rigorous hemodynamic surveillance is warranted, and more intensive therapeutic interventions may become necessary to manage these potential complications (44, 70) (Table 4). 

**Table 4.** Prevalence and Severity of Adverse Effects with Ketamine, Dexmedetomidine, and a Combined Regimen for Cesarean Section Anesthesia

Dexmedetomidine	Combination (Ketamine + Dexmedetomidine; %)	Prevalence (%)	Severity (%)		
**Psychomimetic effects (hallucinations, agitation)**	Common (20 - 30)	Rare (< 1)	Moderate (10 - 15)	15 - 30	Moderate
**Hypertension**	Common (15 - 25 at high doses)	Rare (< 5)	Low (5 - 10)	15 - 25	Mild - moderate
**Tachycardia**	Common (20 - 30 at high doses)	Rare (< 5)	Low (5 - 10)	20 - 30	Moderate
**Bradycardia**	Rare (< 1)	Common (10 - 15)	Moderate (10 - 20)	10 - 15	Moderate
**Hypotension**	Rare (< 5)	Common (10 - 20)	Moderate (15 - 25)	10 - 20	Moderate
**Respiratory depression**	Rare (< 1)	Rare (< 1)	Low (2 - 5)	< 5	Severe
**Nausea/vomiting**	Common (15 - 30)	Low (5 - 10)	Moderate (10 - 20)	15 - 30	Mild
**Dry mouth**	Rare (< 1)	Common (20 - 30)	Moderate (15 - 25)	20 - 30	Mild
**Laryngospasm**	Rare (< 1)	Not reported	Rare (< 1)	< 1	Severe
**Emergence agitation**	Common (10 - 20)	Not reported	Low (5 - 10)	10 - 20	Moderate

## 7. Conclusions

Ketamine, due to its significant analgesic and hemodynamic stabilization capabilities, presents a valuable option for managing intricate obstetric scenarios, notably in patients diagnosed with pre-eclampsia or obesity. Conversely, dexmedetomidine provides unique sedative and anxiolytic effects without compromising respiratory function, positioning it as a beneficial adjunct to regional anesthesia techniques. The synergistic application of these two pharmacological agents facilitates a balanced anesthetic strategy, leading to a reduction in opioid utilization and a consequent minimization of undesirable side effects. As previously elucidated, the pharmacogenetic profile of these medications significantly influences inter-individual variability in patient responses, thereby emphasizing the importance of tailored anesthetic protocols. Current evidence academically substantiates the efficacy of ketamine and dexmedetomidine as valuable adjuncts in CS anesthesia. These agents offer the benefits of opioid-sparing analgesia and the maintenance of hemodynamic stability. Current recommendations suggest the administration of low-dose IV ketamine (0.1 to 0.3 mg/kg) and dexmedetomidine (0.5 μg/kg). However, it is crucial to acknowledge the role of pharmacogenetic variability, which necessitates a personalized approach to drug dosage to optimize patient outcomes. To further refine clinical guidelines, additional RCTs are warranted to focus on comparing neuraxial and general anesthesia protocols in the context of CS to provide a more robust evidence base for anesthetic management.

## 8. Limitations of This Narrative Review

As a narrative review, this work inherently carries certain limitations. Notably, it does not include a formal quantitative meta-analysis, which is a standard component of systematic reviews aimed at statistically pooling data. While a structured search was performed, the qualitative nature of the synthesis means that the findings represent a broad overview rather than a statistically weighted conclusion from homogenous studies. Furthermore, without a formal risk of bias assessment, we were unable to formally evaluate the internal validity of individual studies included in the narrative. Furthermore, this narrative review does not employ a formal evidence grading system (e.g., GRADE), which means that the strength of the recommendations or the overall quality of the underlying evidence cannot be systematically quantified. Consequently, the clinical considerations provided are based on a comprehensive qualitative synthesis of the literature rather than a formally graded evidence profile.

## Data Availability

The dataset presented in the study is available on request from the corresponding author during submission or after publication.
